# Supplementary material for: Resolving the full spectrum of human genome variation using Linked-Reads
Source: Genome Res. 2019 Apr;29(4):635–45. doi: 10.1101/gr.234443.118 (PMC6442396; doi:10.1101/gr.234443.118)
Supplement: Supplemental Material [file supp_gr.234443.118_Supplemental_longranger-2.2.zip › longranger-2.2.tar.bz2!/lr2.2-public/lib/stan/cmdstan/stan/doc/api/html/classstan_1_1gm_1_1arg__adapt__engaged-members.html]

Stan: Member List


|  |  |
| --- | --- |
|  | Stan  2.5.0  probability, sampling & optimization |


- Main Page
- Related Pages
- Namespaces
- Classes
- Files

- Class List
- Class Index
- Class Hierarchy
- Class Members

All Classes Namespaces Files Functions Variables Typedefs Enumerations Enumerator Friends Macros Pages

- stan
- gm
- arg\_adapt\_engaged

stan::gm::arg\_adapt\_engaged Member List

This is the complete list of members for stan::gm::arg\_adapt\_engaged, including all inherited members.

|  |  |  |
| --- | --- | --- |
| \_bad\_value | stan::gm::singleton\_argument< T > | protected |
| \_constrained | stan::gm::singleton\_argument< T > | protected |
| \_default | stan::gm::valued\_argument | protected |
| \_default\_value | stan::gm::singleton\_argument< T > | protected |
| \_description | stan::gm::argument | protected |
| \_good\_value | stan::gm::singleton\_argument< T > | protected |
| \_name | stan::gm::argument | protected |
| \_validity | stan::gm::singleton\_argument< T > | protected |
| \_value | stan::gm::singleton\_argument< T > | protected |
| \_value\_type | stan::gm::valued\_argument | protected |
| arg(const std::string name) | stan::gm::argument | inlinevirtual |
| arg\_adapt\_engaged() | stan::gm::arg\_adapt\_engaged | inline |
| argument() | stan::gm::argument | inline |
| argument(const std::string name) | stan::gm::argument | inline |
| compute\_indent(const int depth) | stan::gm::argument | inline |
| description() const | stan::gm::argument | inline |
| find\_arg(std::string name, std::string prefix, std::vector< std::string > &valid\_paths) | stan::gm::singleton\_argument< T > | inlinevirtual |
| help\_width | stan::gm::argument | protected |
| indent\_width | stan::gm::argument | protected |
| is\_default() | stan::gm::singleton\_argument< T > | inlinevirtual |
| is\_valid(T value) | stan::gm::singleton\_argument< T > | inlineprotectedvirtual |
| name() const | stan::gm::argument | inline |
| parse\_args(std::vector< std::string > &args, std::ostream \*out, std::ostream \*err, bool &help\_flag) | stan::gm::singleton\_argument< T > | inlinevirtual |
| print(std::ostream \*s, const int depth, const std::string prefix) | stan::gm::valued\_argument | inlinevirtual |
| print\_help(std::ostream \*s, const int depth, const bool recurse=false) | stan::gm::valued\_argument | inlinevirtual |
| print\_valid() | stan::gm::singleton\_argument< T > | inlinevirtual |
| print\_value() | stan::gm::singleton\_argument< T > | inlinevirtual |
| probe\_args(argument \*base\_arg, std::stringstream &s) | stan::gm::singleton\_argument< T > | inlinevirtual |
| set\_value(const T &value) | stan::gm::singleton\_argument< T > | inline |
| singleton\_argument() | stan::gm::singleton\_argument< T > | inline |
| singleton\_argument(const std::string name) | stan::gm::singleton\_argument< T > | inline |
| split\_arg(const std::string &arg, std::string &name, std::string &value) | stan::gm::argument | inlinestatic |
| value() | stan::gm::singleton\_argument< T > | inline |
| ~argument() | stan::gm::argument | inlinevirtual |


---

[ Stan Home Page ]

*© 2011–2014,
Stan Development Team.*
